# Microstructures and Phases of Ytterbium Silicate Coatings Prepared by Plasma Spray-Physical Vapor Deposition

**DOI:** 10.3390/ma13071721

**Published:** 2020-04-07

**Authors:** Jie Xiao, Qian Guo, Liangliang Wei, Wenting He, Hongbo Guo

**Affiliations:** 1 School of Materials Science and Engineering, Beihang University, No. 37, Xueyuan Road, Beijing 100191, China; xiaojie@buaa.edu.cn (J.X.); guoqian@buaa.edu.cn (Q.G.); weill@buaa.edu.cn (L.W.); 2 Key Laboratory of High-temperature Structural Materials &Coatings Technology (Ministry of Industry and Information Technology), Beihang University, No. 37, Xueyuan Road, Beijing 100191, China

**Keywords:** ytterbium silicate, plasma spray physical vapor deposition (PS-PVD), microstructure, phases

## Abstract

Ytterbium silicate coatings were deposited on SiCf/SiC ceramics matrix composite (CMC) substrates by plasma spray-physical vapor deposition (PS-PVD), and the microstructures and phase constituents of the coatings were studied. The results show that the Yb_2_SiO_5_ coating prepared with high power and low pressure (65 kW/2 mbar) had quasi-columnar structure, mainly deposited from the vapor phase, whereas the coating prepared with low power and high pressure (40 kW/10 mbar) had a typical layered structure, mainly deposited from the liquid phase. The deposition efficiency of parameter A (~2 μm/min) was also significantly lower than that of parameter B (~20 μm/min). After annealing at 1300 °C for 20 h, the coating prepared by 65 kW/2 mbar was mainly composed of ytterbium disilicate phase (77.2 wt %). The coating also contained some silicon-rich phase. The coating prepared by 40 kW/10 mbar basically consisted of ytterbium monosilicate (63.6 wt %). In addition, a small amount of silicon-rich phase and ytterbium-rich phase were also present in the coating. Accompanied with calculation results by the FactSage software, the cause of deviations in phase compositions was analyzed.

## 1. Introduction

In order to meet the service requirements of next-generation aircraft engines with a high thrust-to-weight ratio, the application of ceramic matrix composites (CMCs) to thermal structural parts of engines is being increasingly considered for replacing the existing superalloys [1,2]. In a hot, dry, and oxygen-containing atmosphere, CMCs can produce a stable and dense silicon oxide protective layer on the surface to inhibit further oxidation. Once CMCs are exposed to fast-flowing water-vapor-rich gasses, silicon oxide reacts easily with water vapor, forming gaseous silicon hydroxide and therefore causing degradation of CMC components [3]. This reaction results in a serious drop in the thermal and mechanical properties of the CMCs. A protective coating on the surface of the CMCs (namely, an environmental barrier coating, EBC), is therefore necessary to protect CMCs from environmental corrosion and improve both the service life and service temperature of CMCs [4].

Through many years of development, EBCs have evolved from the first single-layer of mullite to the advanced three-layer structure, including Si/mullite/rare earth silicate [5,6]. In a service environment, the silicon layer prevents oxidation of CMCs substrate by forming a layer of silicon oxide. The top ceramic layer (rare earth silicate) is mainly used to block the entry of water vapor, and the mullite layer can reduce the mismatch of coefficients of thermal expansion (CTEs) and alleviate thermal stresses across the layers [7]. Among rare earth silicates, Yb_2_SiO_5_ (YbMS) and Yb_2_Si_2_O_7_ (YbDS) are the most promising EBC materials since they have several advantages [8]. These include a high melting point, low volatility [9], excellent mechanical properties, low thermal conductivity, and good phase structure stability above 1400 °C [10,11,12,13,14,15,16]. Some thermal-mechanical properties of bulk ytterbium silicate materials are shown in Table 1 [17].

Compared with YbDS, YbMS exhibits better resistance to water vapor corrosion and CMAS (CaO_2_–MgO–Al_2_O_3_–SiO_2_) attack [6,13]. However, Xu Yue et al. found that the CTE of YbMS (7.2 × 10^−6^ K^−1^) is relatively larger than that of the intermediate mullite layer (5~6 × 10^−6^ K^−1^) [4]. Hence, β-RE_2_Si_2_O_7_ (RE = Sc, Lu, and Yb) and the intermediate mullite layer match well. Therefore, it was suggested that a suitable composite containing RE_2_Si_2_O_7_ and RE_2_SiO_5_ could adjust the CTEs and the water vapor resistance of composites to a balanced level for further prolonging the service life of the above-mentioned tri-layer EBC system [4,18].

Currently, the main preparation process for ytterbium silicate coatings is atmospheric plasma spraying (APS) [5,19]. Normally, APS EBCs contain many pores and semi-melted particles. The gas-tightness of such a typical structure is not sufficient for use as an EBC. Plasma spray-physical vapor deposition (PS-PVD) is an emerging coating preparation technology that can be used to prepare various advanced coatings [20,21], such as columnar structured coatings, as well as thin and dense coatings by depositing gas and/or liquid and/or solid phases [17,22,23,24]. In ytterbium silicate, silicon oxide is easily vaporized during the spraying process [25]. Taking advantage of PS-PVD, it is possible to manufacture dense EBCs consisting of YbDS and YbMS phases.

In this paper, the ytterbium silicate topcoats were prepared using two different PS-PVD parameters. The deposition rate, microstructures, and phase compositions of as-deposited coatings as well as annealed coatings were studied in detail. Additionally, the component deviation was analyzed by FactSage software, which can provide some guidance for preparing EBC using PS-PVD in the future.

## 2. Materials and Methods

### 2.1. Coating Spraying

First, the Si and mullite layers were coated by APS (Chinese Academy of Agricultural Mechanization Sciences, Beijing, China) on SiC_f_/SiC CMC substrates (10 mm × 20 mm × 4 mm), the APS parameters are presented in Table 2. Ytterbium silicate top-coats were deposited on the Si/mullite coating by PS-PVD (Medicoat AG, Mägenwil, Switzerland). The PS-PVD parameters are presented in Table 3, in which the main variables are the net power from 65 kW (sample A) to 40 kW (sample B) and the vacuum pressure from 2 to 10 mbar. The as-sprayed coatings were then annealed at 1300 °C for 20 h in a vacuum furnace.

**Figure 1:**
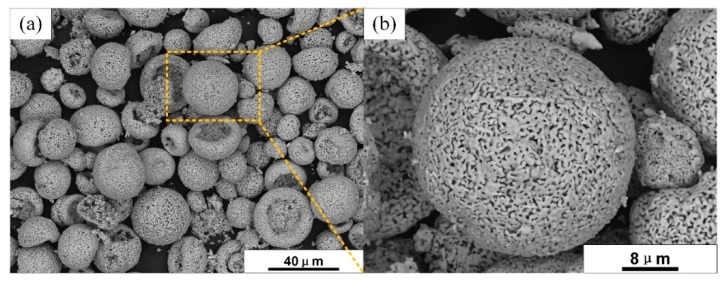
Images of ytterbium silicate feedstock powders at (**a**) low magnification; (**b**) high magnification.

The ytterbium silicate powder grade was A-20170522 (Guangzhou Research Institute for Non-ferrous Metals, Guangzhou, China), and the powder particle size D50 was 10–30 μm. The images of ytterbium silicate powder are presented in Figure 1. XRD patterns of the starting powders (mullite and Yb_2_SiO_5_) have been given in our previous article [26], wherein peaks in the XRD pattern of Yb_2_SiO_5_ match the standard powder diffraction files (PDFs) perfectly without additional peaks, which means that the synthesized Yb_2_SiO_5_ possessed high phase purity.

### 2.2. Characterization

Phase compositions of both as-sprayed and annealed samples were identified by X-ray diffraction (XRD, D/Max 2200PC, Cu/Kα, Rigaku, Tokyo, Japan) at a scanning 2θ speed of 6°/min. The XRD results were analyzed using Jade software (Version 6.5). The crystallinities of samples (*C*) were calculated by Formula 1:

(1) $$C=\frac{\sum I_{Ci}}{\sum I_{Aj}+\sum I_{Ci}}\times 100\%\;$$

where *I_Ci_* is the diffraction intensity of each crystallization peak and *I_Aj_* is the diffraction intensity of each amorphous peak. The *i*-th phase weight percentages (*Wi*) were calculated by Formula 2 [27]:

(2) $$W_{i}=\frac{\frac{I_{i}}{K^{i}}}{\sum \frac{I_{i}}{K^{i}}}\times 100\%$$

where *I_i_* is the strongest peak intensity of the *i*-th phase and *Ki* is the RIR (Reference Intensity Ratio) value of the *i*-th phase.

Microstructures and constituents of the as-sprayed and annealed coatings were characterized by a scanning electron microscope (SEM, Gemini SEM 300, Zeiss, Oberkochen, Germany) equipped with an energy-dispersive spectrometer (EDS).

## 3. Results and Discussion

### 3.1. Microstructures and Compositions of As-Sprayed Coatings 

**Figure 2:**
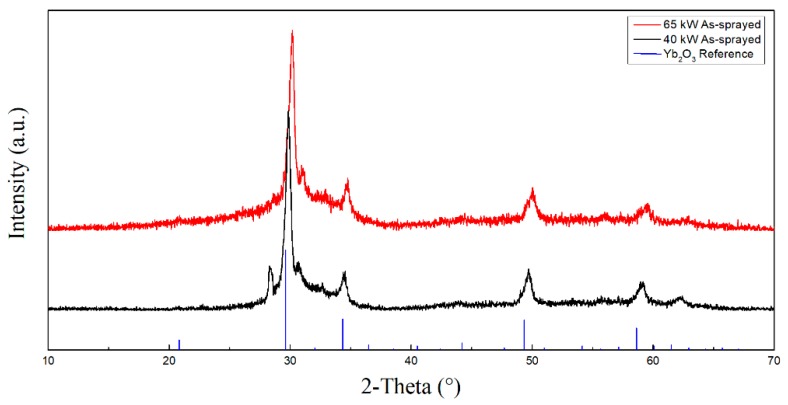
XRD patterns of as-sprayed ytterbium silicate top-coats.

Figure 2 shows the XRD patterns of the as-sprayed ytterbium silicate top-coats. It can be seen that the as-sprayed coatings of both sample A and sample B were mainly Yb_2_O_3_ phase. Besides, both XRD patterns have an amorphous diffuse scattering peak at about 30°, indicating amorphous phases in the coatings, and the crystallinities of sample A and sample B were 38.22% and 59.67%, respectively. During the preparation of the coatings, the powder particles were heated and melted or even vaporized in the plasma plume. Due to the large temperature difference between the substrate and the particles, the melted or vaporized particles were rapidly cooled and solidified on the surface of the substrate; therefore, amorphous structures appeared in the coatings.

**Figure 3:**
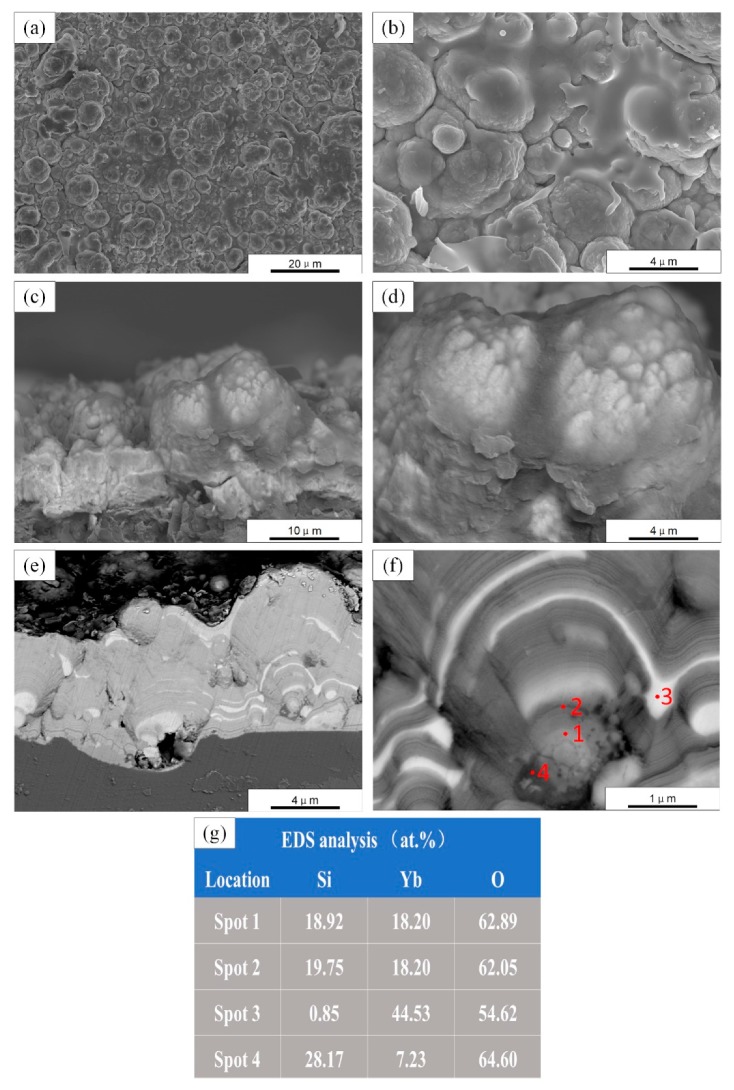
SEM micrographs of as-sprayed ytterbium silicate top-coats prepared by parameter set A: (**a**,**b**) Surface morphology; (**c**,**d**) Fracture morphology; (**e**,**f**) Cross section by back-scattered electron; and (**g**) EDS analysis of four spots in (**f**).

The net power in PS-PVD is much higher than in APS, which can melt well or even evaporate ceramic powder, depending on the size and material of the powder. Due to the high spraying power and low vacuum pressure of parameter set A, the spraying powder could be well gasified, and sample A was mainly deposited from vapor phase. The surface morphology of sample A is shown in Figure 3a. It can be seen that there were many column caps on the coating surface, and the diameter of the column caps was 5–10 μm. At a higher magnification, some non-gasified splats can be seen spreading on the surface of the column caps, which indicates that the coating formed by gas–liquid mixed deposition. The fracture morphologies of sample A are shown in Figure 3c,d. It can be seen that the thin coating is columnar. Figure 3e,f shows cross-sectional micrographs (back-scattered electron) of sample A. During the deposition process, the growth of the stigma can be interrupted by deposition of liquid droplets. However, the growth of column caps would resume due to the deposition of vapor phase. The cross-sectional microstructure further demonstrates that coating A was dominated by vapor-phase deposition. By the higher magnification in Figure 3f, layers with different contrast along the growth direction of the coating can be observed, which means that the deposition process was not uniform. The average thickness of the ytterbium silicate top-coats prepared by parameter A was ~10 μm. Through EDS analyses as shown in Figure 3g, the phase composition of the regions represented by spot 1 and spot 2 are close to Yb_2_Si_2_O_7_, and there are brighter Yb-rich regions (spot 3) and darker Si-rich regions area (spot 4).

**Figure 4:**
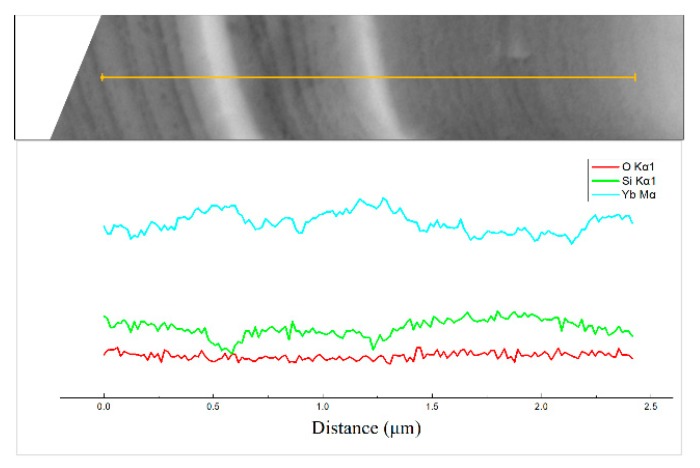
EDS line scan result of the as-sprayed ytterbium silicate top-coat prepared by parameter set A.

The compositional fluctuation within the ytterbium silicate top-coats of sample A, assessed by using a line scan, is shown in Figure 4. The results indicate that the darker contrast has a higher Si content and a lower Yb content. In comparison, the whiter contrast has a higher Yb content and a lower Si content. These are consistent with the EDS analysis in Figure 3f.

**Figure 5:**
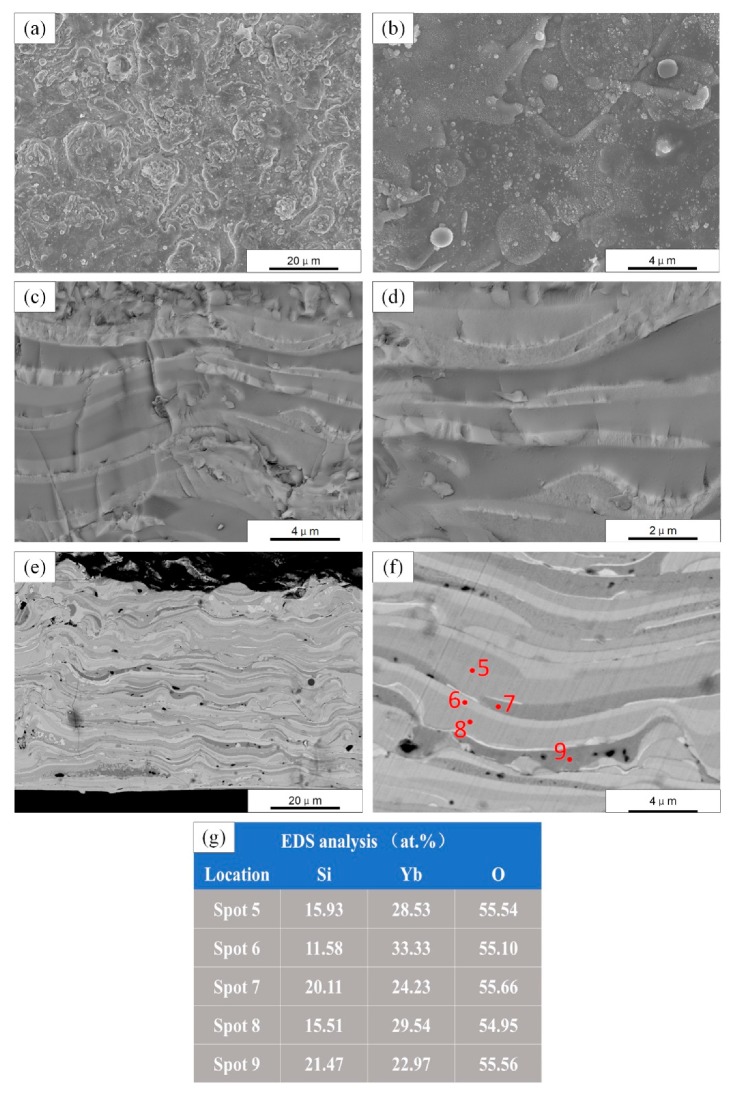
SEM micrographs of as-sprayed ytterbium silicate top-coats prepared by parameter set B: (**a**,**b**) Surface morphology; (**c**,**d**) Fracture morphology; (**e**,**f**) Cross section by back-scattered electron; and (**g**) EDS analysis of five spots in (**f**).

Due to the relatively lower spraying power (40 kW) of parameter set B, the powder could not be gasified in large quantities. The surface morphology of coating B is shown in Figure 5a,b. Completely molten droplets were sputtered and spread rapidly on the surface of the substrate, forming splats. Some particles also can be seen in Figure 5b, indicating the presence of partially melted feedstock during the deposition of the coating. The fracture morphologies of sample B are shown in Figure 5c,d. Obviously, the coating had a layered structure, which means that the coating deposition was dominated by liquid droplets. Figure 5e,f shows the cross-sectional micrographs (back-scattered electron) of sample B. The thickness of the banded structure was about 1–2 μm, and the average thickness of the top-coats prepared by parameter set B was ~60 μm. Referring to the deposition rate of parameter set A (~2 μm/min) the parameter B (~20 μm/min) is significantly higher. This might be because the plasma jet in such a low-pressure chamber (2 mbar) was over-expanded, and a large amount of vaporized phase was taken away along with the plasma gases by the vacuum pump, resulting in a relatively low deposition rate on the substrate.

The deposited coating had a distinct banded structure with light and dark contrast changes. This is because during the PS-PVD spraying process, the heating degree of each droplet is different, and the temperature of each droplet is different after heating, resulting in the difference of SiO_2_ and Yb_2_O_3_ evaporation rates in the droplets. As a result, the final composition deviation was also different. Composition analysis by EDS was performed on Figure 5f, and the results are shown in Figure 5g. Bands with different contrast have different Yb/Si atomic ratios: from 3:1 in the lighter region to 1:1 in the darker region. The Yb/Si atomic ratio of the initial spray powder was 2:1. This indicates that the composition of the droplets deviated during the flight in the plasma plume.

**Figure 6:**
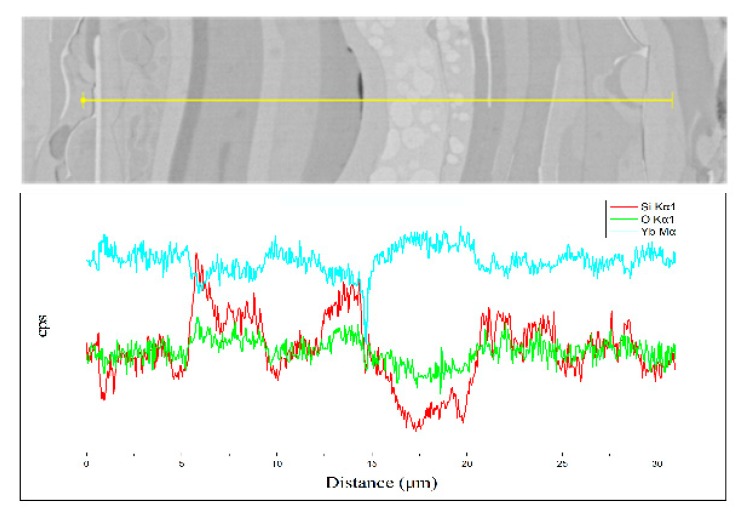
EDS line scan of the as-sprayed ytterbium silicate top-coats prepared by parameter set B.

Again, the compositional fluctuation within sample B as assessed using a line scan is shown in Figure 6. The results are consistent with the analysis of EDS in Figure 5f.

### 3.2. Microstructure and Composition of Annealed Coatings

**Figure 7:**
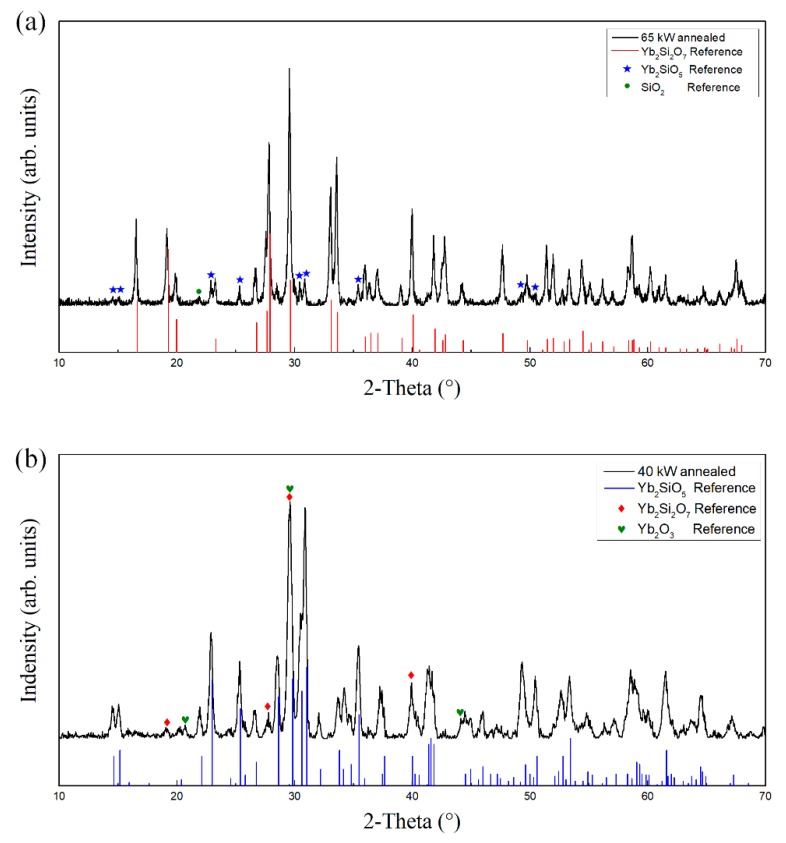
XRD patterns of annealed ytterbium silicate top-coats prepared by (**a**) 65 kW and (**b**) 40 kW.

Figure 7 shows the XRD patterns of the annealed ytterbium silicate top-coats. After annealing, the two coatings were fully crystallized. According to the XRD analyses, the main component of sample A (Figure 7a) after annealing was YbDS, along with a small amount of YbMS and SiO_2_ phases, the percentages of YbDS, YbMS, and SiO_2_ were 77.2, 15.5, and 7.3 wt %, respectively. In contrast, the main component of sample B (Figure 7b) was YbMS, but there was also a small amount of YbDS and Yb_2_O_3_ phases; the percentages of YbMS, YbDS, and SiO_2_ were 63.6, 29.7, and 6.7 wt %, respectively.

**Figure 8:**
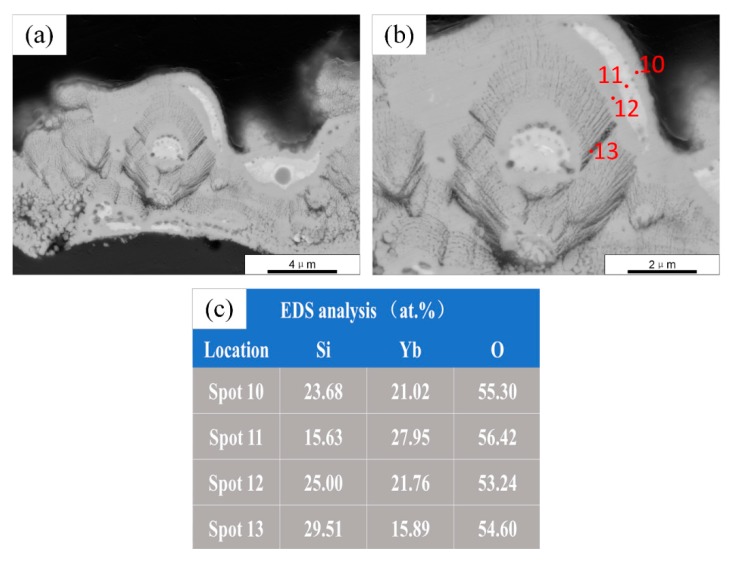
SEM micrographs of annealed ytterbium silicate top-coats prepared by parameter set A: (**a**,**b**) Cross section by back-scattered electron; and (**c**) EDS analysis of four spots in (**b**).

**Figure 9:**
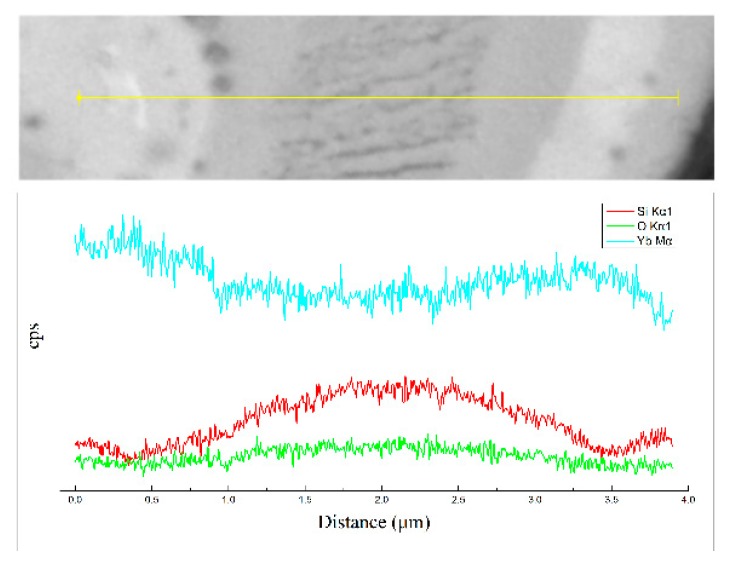
EDS line scan of annealed ytterbium silicate top-coats prepared by parameter set A.

Figure 8a shows the cross-sectional micrograph (back-scattered electron) of sample A after annealing. It can be seen that the inner structure of the coating was more uniform than before annealing, showing three kinds of contrast. The results of EDS line scan are shown in Figure 9, which show that the relative contents of Si and Yb varied within the measured range. Four different points were selected (Figure 8b) for EDS analyses, and corresponding results are shown in Figure 8c. The phase composition of the middle contrast area (Spot 10 and Spot 12) with the largest proportion is YbDS, the phase composition of the brighter contrast area (Spot 11) is basically the same as that of YbMS, and the phase composition of the darker contrast area (spot 13) is Si-rich phase. The EDS analyses are consistent with the results of XRD.

**Figure 10:**
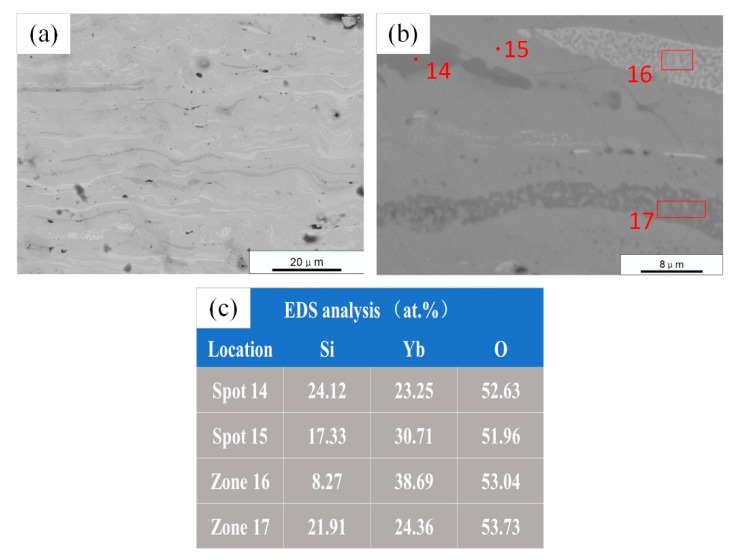
SEM micrographs of annealed ytterbium silicate top-coats prepared by parameter set B: (**a**,**b**) Cross section by back-scattered electron; and (**c**) EDS analysis of four spots in (**b**).

**Figure 11:**
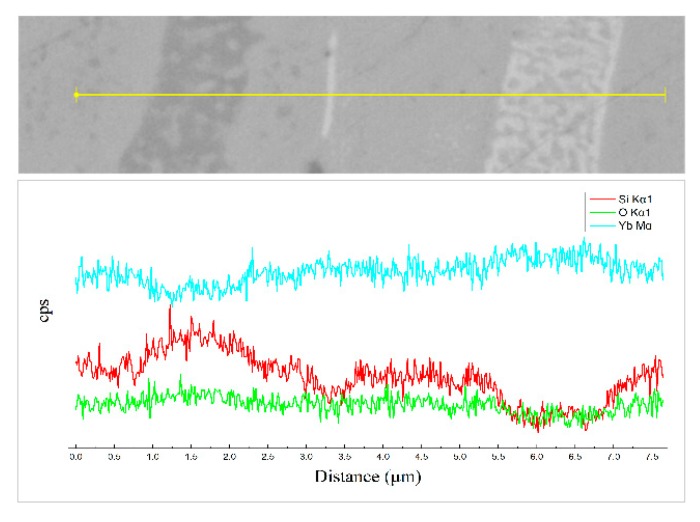
EDS line scan of the annealed ytterbium silicate top-coats prepared by parameter set B.

**Figure 12:**
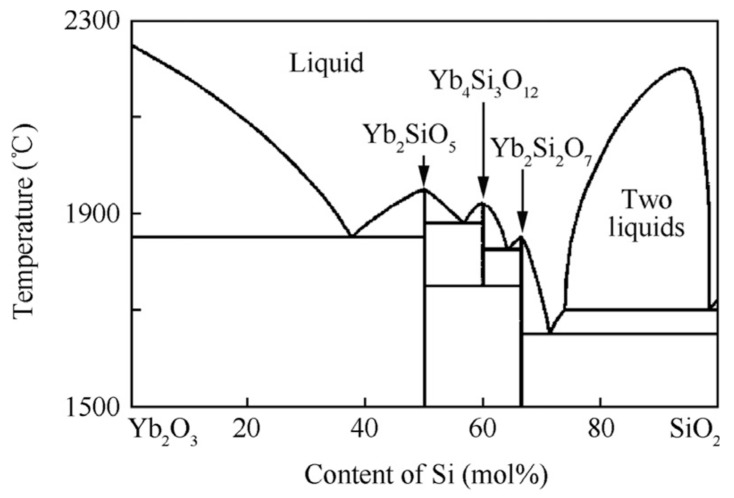
SiO_2_-Yb_2_O_3_ pseudo-binary phase portrait.

The cross-sectional micrographs (back-scattered electron) and EDS analyses of the annealed sample B are shown in Figure 10. The structure of the annealed coating in Figure 10a is more uniform than the as-sprayed one, and there are mainly three different degrees of contrast. Figure 10b shows a high-magnification micrograph wherein the areas with medium contrast are the main components, the areas with whiter contrast are mainly two-phase mixtures, and the areas with darker contrast are both single-phase and two-phase mixtures of contrast. The compositional fluctuations were analyzed using a line scan, as shown in Figure 11. As with sample A, the content of Si and Yb varied significantly with the contrast. In Figure 10b, two representative points and two regions were selected for EDS analyses. According to the EDS results, the main component of the coating B after annealing was YbMS (grey contrast area), and there was also a small amount of YbDS (dark contrast area) and Yb_2_O_3_ (white contrast area). YbDS was distributed in a separate region (spot 14) and a region eutectic with YbMS (Zone 16). Yb_2_O_3_ mainly formed a eutectic (Zone 17) with YbMS. The results of EDS analyses are consistent with the XRD results. The phase composition deviation in different regions was mainly determined by the droplet composition. According to the SiO_2_-Yb_2_O_3_ pseudo-binary phase diagram in Figure 12 [28], when the Yb/Si atom ratio in the droplet was higher than 2:1, a eutectic of Yb_2_O_3_ and YbMS was formed; when the Yb/Si atom ratio in the droplet was between 2:1 and 1:1, YbDS formed a eutectic with YbMS [7].

**Figure 13:**
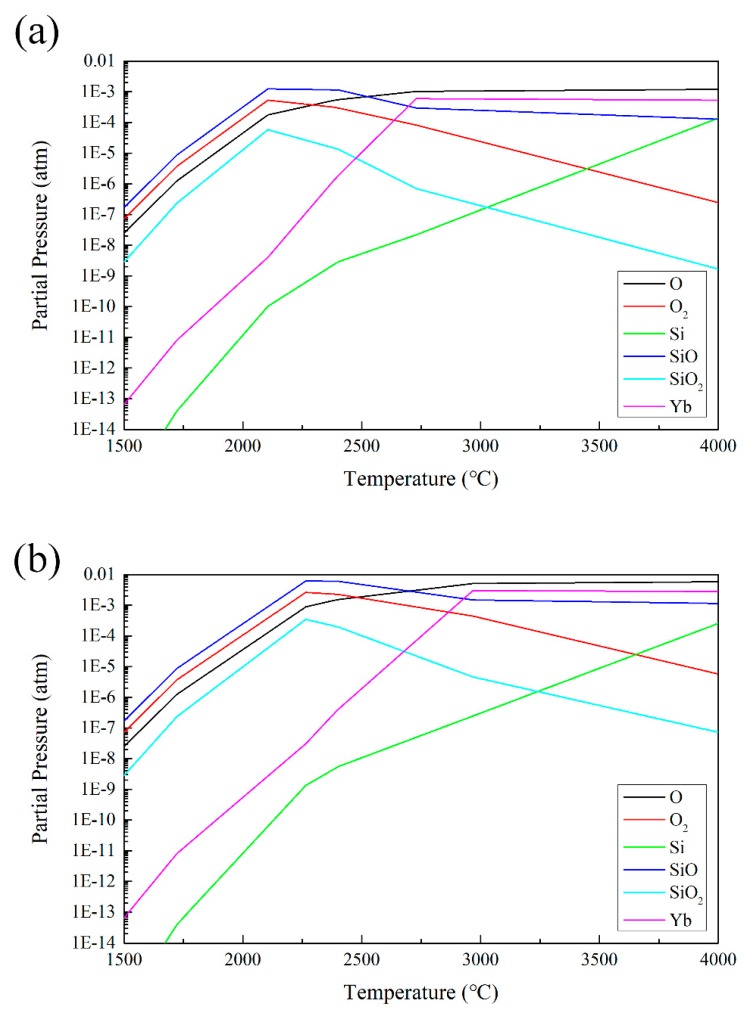
Calculated partial pressure versus temperature plot for primary gaseous species in the Yb–Si–O system in (**a**) 2 mbar and (**b**) 10 mbar. Curves are reported for an equilibrated system.

According to the literature, because of its high saturated vapor pressure, SiO is more volatile in the ytterbium silicate coating prepared by APS during the spraying process. So, the components of the prepared coating were mainly Yb_2_O_3_ and YbMS [5,7,29]. However, as mentioned above, the coatings prepared by PS-PVD—especially sample A—had an Yb/Si atomic ratio that moved in a completely opposite direction to the APS coatings [7]. The Equilib module in FaceSage software (Version 7.3, Education) was used to calculate the vapor pressures of species in equilibrium of a mixed gas formed by Yb_2_O_3_ + SiO_2_ at 2 mbar and 10 mbar, respectively. As shown in Figure 13, the gas partial pressure was a function of temperature.

In PS-PVD, the spraying process can be abstracted into four steps: the powder is heated in the plasma gun; the heated particles enter the low-pressure compartment; the particles fly in the plasma plume; and then the particles impact on the substrate. Meanwhile, deposition of the coating occurs. When the heated particles enter the low-pressure environment, compared with the calculation results under 1 atm pressure [5], as the vacuum pressure decreases, the complete gasification temperature of YbMS gradually decreases. At the same time, the power of sample A (65 kW) was much higher than for APS, which makes YbMS much easier to be heated to a sufficiently high temperature, making it easy to gasify and decompose when entering the low-pressure environment, and generate Yb gaseous monoatoms. The power of sample B (40 kW) could not completely vaporize the YbMS powder, and the majority of the YbMS powder could only be melted into droplets. When the droplets were flying in the plasma plume, the high partial vapor pressure of SiO at elevated temperatures resulted in a substantial loss of silicon from the droplet during flight in the plasma plume [7]. For Yb_2_O_3_, as shown in Figure 13, unlike SiO and SiO_2_, it had no gas phase, and directly decomposed into Yb and O during gasification. Because of the high temperature of plasma plume, once gasified, it is difficult for Yb to react with O to produce liquid Yb_2_O_3_. Therefore, during vapor deposition (the last step), the sublimation temperature has an important effect on the deposition efficiency of Yb. The phase change process of Yb was also calculated from the FactSage software. At 10 mbar, the Yb phase change and reaction with O_2_ were according to Equation (3) and Equation (4):

(3) $$\mathrm{Yb}\left(g\right)\overset{763.46\;{}^{\circ}C}{\to }\mathrm{Yb}\left(s,\;\mathrm{bcc}\right)$$

(4) $$\mathrm{Yb}\left(s,\;\mathrm{bcc}\right)+O_{2}\to \;{\mathrm{Yb}}_{2}O_{3}(s)$$

and at 2 mbar, the phase change and reaction are performed according to Equation (5) and Equation (6):

(5) $$\mathrm{Yb}\left(g\right)\overset{673.51\;{}^{\circ}C}{\to }\mathrm{Yb}\left(s,\;\mathrm{fcc}\right)$$

(6) $$\mathrm{Yb}\left(s,\;\mathrm{fcc}\right)+O_{2}\to \;{\mathrm{Yb}}_{2}O_{3}(s)$$

The calculation results indicate that with decreasing vacuum pressure, the gasification temperature of Yb gradually decreases; at the same time, the PS-PVD power is high and the heating capacity is stronger. Therefore, once YbMS decomposes into Yb atoms, at low chamber pressure, compared with SiO, the Yb atoms will be more difficult to solidify on the substrate, and will be carried away by the gas flow. Thus, the ytterbium silicate coating composition prepared by PS-PVD moved to the direction of decreasing Yb/Si atomic ratio, especially sample A.

## 4. Conclusions

In this paper, ytterbium silicate coatings were prepared by PS-PVD and the microstructure and phase constituents of the coatings before and after annealing were studied. Some conclusions can be drawn as follows:

(1) An ytterbium silicate coating with columnar structure based on vapor deposition could be prepared by high net power (65 kW), whereas a coating with layered structure could be prepared by low net power (40 kW) based on liquid-phase deposition. The two as-sprayed coatings were mainly composed of amorphous phase.
(2) Crystallization of the as-sprayed coatings was completed when the coatings were annealed at 1300 °C for 20 h. The coating sprayed at 65 kW was mainly composed of YbDS and also contained a small amount of YbMS phase and SiO_2_ phase. In contrast, the coating sprayed at 40 kW basically consisted of YbMS. Besides, a small amount of YbDS phase and Yb_2_O_3_ phase were present in the coating.
(3) The calculation results by FactSage software indicate that YbMS powder can be decomposed into single gaseous Yb atoms during the PS-PVD process. Because of low vacuum, Yb gaseous atoms in high-temperature and high-speed gas flow are more difficult to solidify, resulting in a low atomic ratio of Yb/Si in the PS-PVD coatings compared to that of APS coatings.

These results indicate that PS-PVD is one of the most promising technologies to prepare high-performance EBCs. The effects of the coating microstructures on the resistance to high-temperature oxidation and steam corrosion will be the next subject of our work.

## Figures and Tables

**Table 1:** Thermal-mechanical properties of bulk Ytterbium silicate materials.

	Melting Point	Coefficient of Thermal Conductivity	Coefficients of Thermal Expansion	Elastic Modulus	Hardness
	(°C)	(W/m K)	(×10^−6^ K^−^^1^)	(GPa)	(GPa)
Yb_2_SiO_5_	1950	2.3–1.5 (300–1400 K)	7–8	149	6.4 ± 0.1
Yb_2_Si_2_O_7_	1850	4.6–2.0 (300–1400 K)	3.7–4.5 (800–1600 K)	168	7.3 ± 0.2

**Table 2:** Atmospheric plasma spraying (APS) process parameters.

Parameter	Si	Mullite
Net power/kW	44.4	33.1
Spray distance/mm	100	110
Ar/L/h	2000	2269
H_2_/L/h	80	567
Current/A	600	650
Feed rate/g/min	9.5	12.0
Substrate temperature/°C	1000	1100

**Table 3:** Two different sets of spray process parameters.

Parameter	Sample A	Sample B
Net power/kW	65	40
Spray distance/mm	1000	1000
Ar/L/min	30	30
He/L/min	60	60
Powder feeding rate/g/min	10	10
Powder carrier gas/L/min	10	2
Vacuum pressure/mbar	2	10
Deposition time/min	5	3
